# Genetically engineered mouse models and human osteosarcoma

**DOI:** 10.1186/2045-3329-2-19

**Published:** 2012-10-04

**Authors:** Alvin JM Ng, Anthony J Mutsaers, Emma K Baker, Carl R Walkley

**Affiliations:** 1 St Vincent’s Institute of Medical Research, 9 Princes Street, Fitzroy, VIC, 3065, Australia; 2 Department of Medicine, University of Melbourne, St. Vincent’s Hospital, Fitzroy, VIC, 3065, Australia; 3 Ontario Veterinary College, University of Guelph, 50 Stone Road, Guelph, ON, N1G 2W1, Canada

**Keywords:** Osteosarcoma, p53, Rb, Mouse models

## Abstract

Osteosarcoma is the most common form of bone cancer. Pivotal insight into the genes involved in human osteosarcoma has been provided by the study of rare familial cancer predisposition syndromes. Three kindreds stand out as predisposing to the development of osteosarcoma: Li-Fraumeni syndrome, familial retinoblastoma and RecQ helicase disorders, which include Rothmund-Thomson Syndrome in particular. These disorders have highlighted the important roles of *P53* and *RB* respectively, in the development of osteosarcoma. The association of OS with *RECQL4* mutations is apparent but the relevance of this to OS is uncertain as mutations in *RECQL4* are not found in sporadic OS. Application of the knowledge or mutations of *P53* and *RB* in familial and sporadic OS has enabled the development of tractable, highly penetrant murine models of OS. These models share many of the cardinal features associated with human osteosarcoma including, importantly, a high incidence of spontaneous metastasis. The recent development of these models has been a significant advance for efforts to improve our understanding of the genetics of human OS and, more critically, to provide a high-throughput genetically modifiable platform for preclinical evaluation of new therapeutics.

## Review

### Osteosarcoma

Osteosarcoma (OS) is the most common primary tumour of bone. It is most frequent in children and adolescents with an incidence of 7.3 per 1 million of the population [1]. Although OS is mainly classified as a childhood disease, a second peak of incidence is reported in the elderly population [1]. The majority of OS tumours are situated in the long bones with a small proportion located in the pelvis and axial skeleton [2,3]. OS has a relatively high metastatic rate, with the lung being the most common site of spread.

The current treatment for OS revolves around the use of chemotherapy, radiotherapy and the surgical removal of the tumour. The chemotherapeutic regimen for OS patients combines cisplatin, doxorubicin and high doses of methotrexate [4]. Surgical resection is coupled with limb salvage procedures to remove malignant tissue and minimize the impact on quality of life.

The lack of new therapeutic options for the management of OS has translated to a stagnation of patient outcomes [5,6]. Survival and prognosis rates have remained largely unchanged in two decades despite increased detection and monitoring afforded by advances in clinical imaging modalities [7-9]. Furthermore, there are difficulties associated with the study of OS in humans, such as recruiting sufficient patients to allow clinical insights in trialing new treatment options. A key component to improving patient outcome will be the development and application of faithful experimental models of human OS. Such models can serve as a preclinical platform for the identification of new therapeutic targets and the *in vivo* testing and triaging of those proposed for human trials. Experimentally derived interventions could then be developed in *in vivo* models where therapies can be rigorously evaluated side by side prior to human evaluation. Equally importantly, experimental OS models serve as a means to further understand the genetics and biology of OS with an emphasis on metastatic disease.

### Animal models of osteosarcoma

Robust animal models have the capacity to preclinically evaluate therapeutic interventions derived from the extensive basic research efforts underway in OS. To date, the major species used to deliberately generate experimental OS are the mouse and the rat [10,11]. The lineage and temporal specificity afforded by murine genetic engineering has lead to a rapid increase in the quality and fidelity of murine OS models when compared to the human condition. Spontaneous disease arising in large breed pet dogs is also of note as a model of human OS and is useful to understanding OS in humans and veterinary practice. It is also gaining prominence in the research environment as a validated model of spontaneous OS [12-14].

Rodent models of OS have been established for many decades and were originally generated through the exposure to chemical and radioactive carcinogens. [15-17]. These models demonstrated the principle of high-penetrance OS models that histologically resemble human OS. However, they possessed several caveats regarding their application to preclinical studies. The majority of OS in humans is sporadic, while the carcinogen-induced murine OS are more representative of therapy-induced disease rather than the primary lesions arising in the majority of human OS [18,19]. Radiation induced OS models generally have a longer latency than alternate strategies and can result in a range of non-mesenchymal tumours due to its non-specific nature. Furthermore it has not been clearly defined what genetic lesions occur during the initiation and maintenance of these tumours. Nonetheless, these radiation-induced OS models have yielded robust experimental data and gave rise to valuable reagents such as cell lines to complement human OS studies. Further characterization of these tumours would enable the rational application of these alongside the recently generated tractable genetically engineered models.

### Human hereditary disorders: insight into the genetics of human OS

Rare human hereditary disorders offer powerful insights into genes that play critical roles in human cancer biology *in vivo*. This is because they offer unequivocal evidence of defined genetic lesions and their importance in human disease pathogenesis. There is a cluster of familial syndromes that predispose to the development of OS and are of relevance to understanding the underlying genetics of OS. Li-Fraumeni syndrome, familial Retinoblastoma and RecQ helicase disorders such as Rothmund-Thomson Syndrome (RTS) are caused by germ-line mutations of *P53*, *RB* and *RECQL4* respectively. These three kindreds have a greatly enhanced incidence of OS compared to the general population as documented in a range of clinical studies in affected families. In particular, Li-Fraumeni Syndrome patients are highly prone to develop OS, while OS is the second most common tumour type in Retinoblastoma patients [20-22]. OS tumours are a frequent feature of the tumour spectrum affecting RTS patients, however unlike mutations in p53 and the Rb pathway, RECQL4 mutations are not observed in sporadic OS [23].

A range of approaches has been used to incorporate information from clinical human OS to model the disease in the mouse. In particular, transgenic and germ-line loss of function alleles have demonstrated important roles for p53 mutations in generating experimental OS. More recently, lineage-restricted somatic deletion models that generate high penetrant metastatic disease have been described [24,25]. These models will provide a definitive assessment on the roles of genes in the initiation and maintenance of OS. Furthermore they can be exploited to reveal new therapeutic avenues that can be targeted for the development of new therapies, with a particular emphasis on metastatic disease.

## Human hereditary disorders and osteosarcoma

### Li-fraumeni syndrome (LFS)

Li-Fraumeni syndrome is an autosomal dominant disorder with germ-line heterozygous mutation in *P53*. It is characterized by a predisposition to a range of cancers [26,27]. LFS patients have a highly elevated risk of developing soft tissue sarcoma and osteosarcoma [28], and mutations in the “p53-pathway” are thought to be essential for the formation of human cancer.

Mutations in components of the p53 pathway are found in both familial and sporadic OS. Interestingly, the *P53* allele itself is found to be mutated in human OS, most commonly as missense mutations [29,30]. *P53* mutations are not associated with therapeutic response or metastatic status [31,32]. Other reported lesions in the p53 pathway in human OS include amplification of MDM2 and loss of p19^ARF^[33-37].

### Hereditary retinoblastoma

Patients with familial retinoblastoma possess germline mutations in the *Retinoblastoma* (RB) gene [38]. Rb is a critical co-ordinator of G_1_-S phase cell cycle progression through its interaction with E2F and has been implicated in a wide range of cellular processes [39].

OS represents the second most frequent tumour in this kindred after retinoblastoma itself, with nearly half of all patients developing OS [40]. Most cases of sporadic OS present with modifications in at least one allele in the Rb locus [41,42]. The contribution of therapy to OS development in retinoblastoma patients may be more significant than that occurring in LFS. In particular, OS arising from hereditary retinoblastoma is often located at the site of prior radiotherapy. Studies of radiation induced OS has observed mutation of *P53* and retention of the intact *RB* allele in hereditary retinoblastoma patients [43]. As with the p53 pathway, mutations in the members of the Rb pathway occur frequently in OS with known mutations including amplifications of Cyclin E and CDK4 [44-48].

#### OS mouse models based on p53 and Rb mutations

The majority of murine OS models to date have been developed based on knowledge of the mutation of p53 and Rb pathways in both familial and sporadic human OS. Mice with germline mutations of p53 developed OS, but also succumbed to a wide range of tumours [49,50]. Mice with tumour-associated p53 variants presented with a higher incidence of OS than germ-line p53 null animals, amongst the tumour spectrum these animals develop [51]. Mice with homozygous deletions of *RB* are embryonic lethal and their heterozygous counterparts are not predisposed to OS [50,52]. The role of genetic compensation by other family members is apparent with the Rb related p107 and p130 in certain circumstance [53]. However, neither *p107*^*−/−*^ nor *p130*^*−/−*^ mice (or compound mutants that are viable) have a reported susceptibility to OS and these genes are not frequently mutated in human cancers based on data available through the COSMIC database [54].

The move to conditional lineage-restricted alleles of both *p53* and *pRb* has allowed the development of new and more faithful models of OS. Utilising *Prx1*-Cre, which deletes LoxP flanked alleles in the early budding mesenchymal tissue of the limbs, 22% of mice with *p53* heterozygosity develop OS. Homozygous deletion of *p53* had a three-fold increase in OS occurrence. However, the deletion of *Rb* alone in mesenchymal progenitors failed to produce OS tumours [55]. Interestingly the conditional deletion of both *p53* and *Rb* using *Prx1*-Cre resulted in approximately 70% of animals developing a poorly differentiated soft tissue sarcoma (PD-STS). This result suggests that the cell of origin is strongly influencing the arising tumour phenotype, with primitive multipotential cells favoring the development of PD-STS whilst committed osteoblast precursors give rise to OS at high incidence.

A separate group utilized the same transgenic system and yielded similar results. Over 60% of *Prx1*-Cre-*p53*^*fl/fl*^ mice developed OS, while the homozygous deletion of Rb in isolation again yielded no tumours. The compound deletion of one *Rb* allele with homozygous *p53* deletion increased the OS incidence rate to 92%. However, homozygous deletion of both genes yielded only 18% of OS tumours with a strong preference for hibernomas [56].

Rb has been proposed to have a role in influencing late osteoblast differentiation by interacting with Runx2 [57]. However, the removal of *Rb* alone is not sufficient to induce OS in a number of independent studies. *Rb* mutation does show a profound synergy with *p53* mutation in the induction of experimental OS [24,25]. Similarly, shRNAs that reduced Rb expression in p53-deficient OS cell lines (prior to allografts) gave rise to more aggressive and multilineage tumours [56]. The experimental approaches strongly suggest that mutation on the p53 pathway can serve as an initiating event in OS with mutation in the Rb pathway strongly synergizing in the immortalisation of osteoblastic cells.

### Rothmund Thomson syndrome (RTS) and RecQ disorders

RTS is a rare autosomal disorder that consists of epithelial features (skin atrophy, hyper/hypo-pigmentation), congenital skeletal malformations (leading to short stature), premature ageing and increased malignant disease [58]. Most RTS patients have germ-line mutations in the *RECQL4* DNA helicase [59-63]. RTS patients often present with multiple malignancies. In two separate studies, significant portions of RTS patients developed OS with median ages below 11 yrs [23,64]. Conversely, overexpression of Recql4 was reported in human OS tumours with chromosomal abberations and instabilities in the 8q24 locus, which also contains c-Myc [65,66]. RTS patients with truncating *Recql4* mutations associate with a higher risk of developing OS as compared to non-truncated mutations [67,68].

*RECQL4* is a member of a family of DNA helicases including Bloom (*BLM*) and Werner (*WRN*) helicases, All three members are associated with familial cancer predisposition syndromes with high frequencies of mesenchymal derived tumours, with RTS in particular developing OS at approximately 30% frequency. As an ATP-dependent DNA helicase, Recql4 is recruited at the G_1_ and S phases of the cell cycle and plays a critical role in regulating DNA replication. *Recql4* deficiency in mice is associated with karyotypic abnormalities and increased rates of aneuploidy [69,70]. Strikingly in contrast to *p53* and *Rb* mutations, *Recql4* mutations are not associated with sporadic human OS and appear restricted to familial RTS OS. The failure to find *RECQL4* mutations in sporadic OS raises several questions regarding the nature of the disease and whether it represents a distinct entity or subtype of OS. Further efforts characterizing the RTS- related OS are needed to clarify this and efforts to model RTS mutations in mouse may be informative. The contribution of prior chemotherapy/radiotherapy for other cancers arising in RTS patients may be a confounding factor in RTS-associated OS.

#### Recql4 Mutation in the mouse

Of the familial OS syndromes, the least is known about the role of *Recql4*. The expression of *Recql4* shares an inverse relationship with *Rb*, although telomere-lengthening activities are enhanced in cells lacking both genes [71,72]. Interestingly, *Recql4* expression plays a role in osteoblast proliferation but its reduction is reported to be needed for full differentiation [73].

The attempts at modeling of Recql4 deficiency in mice has led to confounding results. Three non-conditional alleles have been reported. The first allele replaced exons 5 through 8 with a LacZ cassette. The homozygous deficient animals were reported as very early embryonic lethal between embryonic days 3–6 [74]. The second reported allele involved deletion of exon 13. The homozygous mutants were viable but exhibited severe growth retardation and multiple abnormalities and 95% of the mice died within 2 weeks of birth [75]. Hetrozygous *Recql4* mutants were viable and had a decreased bone mass [73]. The third allele involved replacement of part of exon 9 through to exon 13 with a PGK-Hprt mini gene cassette [76]. These mice were viable and homozygous *Recql4* deficient animals presented with a range of defects reminiscent of the human RTS alleles. Approximately 16% of mice with homozygous *Recql4* mutations died within 24hrs of birth. 5.8% of animals displayed skeletal defects of the animals that survived past 24hrs. Cancers were detected in 5% of *Recql4−/−* animals in an aged cohort of 100 animals compared to 43 age matched controls, and of these 2 animals developed OS and 3 animals developed lymphoma. This low rate of tumour formation contrasts with the clinical presentation of RTS. The development and characterization of new targeted alleles will be needed to resolve the role of Recql4 in the initiation and maintenance of OS.

### Werner & bloom syndromes

Werner syndrome is characterized by premature ageing and cancer predisposition that occurs during adolescence, whereas Bloom syndrome is characterized by short statures and photosensitive skin [77]. Both disorders are inherited in an autosomal recessive manner, and are attributed to germ-line mutations of the *WRN* and *BLM* genes respectively.

BLM plays a major role in maintaining genomic stability in cells [78]. Likewise, WRN acts against DNA breakages during chromatin structural modifications [79]. It is interesting to note that the expression of BLM and WRN is induced by the loss of Rb. Also, cells that lack the normal expression of all 3 genes presented with enhanced telomere lengthening [71,72]. When treated with chemotherapeutics, cells that were deficient for BLM or WRN had decreased cell proliferation with impaired cell viability [80].

Werner Syndrome patients present with a range of cancers including OS [81,82]. Similarly, patients with Bloom Syndrome are predisposed to various cancers, coupled with an early onset of these tumours [83,84]. As for RTS, the relevance of these mutations to sporadic OS is also unclear and further work is needed to clarify the relationship between these OS and their sporadic counterpart.

#### BLM & WRN mouse models

Genetically engineered mice that habour null mutations of *BLM* were generated by 3 separate groups. Mice with homozygous deletion of *BLM* were embryonic lethal by day 13.5 and presented with an increased level of apoptosis and anaemia [85]. However, viable BLM-null mice were generated with the removal of neomycin plasmid sequence, of which 30% of these mice presented with a wide spectrum of spontaneous tumours [86]. Heterozygous mutant mice were also viable, with a predisposition to develop tumours [87].

Mice with homozygous deficiency for *WRN* were viable and developed tumours by 2 years of age. Interestingly, the combined deletions of *p53* and *WRN* in mice resulted in various soft tissue sarcomas, where half of these mice developed tumours by 3 months of age [88]. However, its strongest link to OS was evident when *WRN* and Telomerase RNA Component (*Terc*) deficiency were combined in mice, with 50% of these mice developing OS [89]. Of note, these were not lineage-restricted alleles suggesting that these pathways co-operate specifically in osteoblasts and strongly synergise in the development of OS.

### Paget’s Disease and p62

Paget’s disease of the bone is characterized by abnormalities in bone growth and destruction, resulting in limb deformities [90]. It is autosomal dominant in nature, and affects mainly adults over the age of 55 [91,92]. It is also often asymptomatic until patients present with fracture or bone pain [93].

Sequestosome1 (SQSTM1) is the only gene currently identified and associated with Paget’s disease of the bone [94]. Also known as p62, this gene contributes to autophagy and removal of abnormal cells [95]. Interestingly, p62 expression needs to be repressed to suppress tumourigenesis [96].

The fraction of patients with Paget’s disease presenting with OS does not exceed 1% [97-101]. This cohort coincide with the second peak of OS incidence rates in the elderly [1,102]. The survival rate of Paget’s disease-associated OS is 5% at 5 years [103].

#### Insights from p62 mouse models

Two separate groups generated transgenic mice that possessed the p62 mutation present in patients with Paget’s disease. There were conflicting results with regards to the histological bone features. However, mice from both groups presented with increased osteoclasts in response to RANKL stimulation, reminiscent of Paget’s disease patients [104,105]. No OS was reported in these mice.

### Other genes associated with osteosarcoma

A range of other genes have been implicated in OS pathogenesis based on studies of human OS samples and cell lines (Table 1). These mutations appear to be cooperative to the defects in the p53 and Rb pathways. Their involvement in OS pathogenesis is also supported by evidence derived from a range of genetically engineered mouse approaches.

**Table 1:** **Additional genes implicated inosteosarcoma (not discussed intext)**

**Gene**	**Human genetic disorder?**	**Gene function / Relevanceto cancer**	**OS penetrance? OS relevance?**	**Mouse model generated?**
p14^ARF^	No	Encoded by the CDKN2a locus; Binds to MDM2-p53 complex to prevent p53 degradation [106]	Ectopic expression in OS cells increases chemo-apoptotic sensitivity [107]; Alterations of p14 genes detected in OS tumour samples [108], which its expression is inverse of p53 [109]; methylation of p14 is linked to poor survival rates for OS patients [110].	Mouse null for the CDKN2a and CDKN2b developed soft-tissue sarcomas [111]
p16^INK4a^	No	Encoded by the CDKN2a locus; CDK4 inhibitor; Member of the RB pathway	Loss of p16 expression in OS tumours with gene deletion detected [44,108,112,113]. Loss of expression in pediatric OS is linked to poor survival [114]; Coexpression with Rb is linked to OS tumour relapse [109].	Mesenchymal stem cells from p16 null mice with overexpressed cMYC developed OS tumours [115]; p16 null mice are larger than wildtype counterparts, and developed soft-tissue sarcomas among other tumour types [116]
p21^CIP1^/ CDKN1a	No	Member of p53 pathway; Cell cycle regulator at G1 phase; Contributes to DNA replication & repair	Overexpression resulted ion growth arrest in OS cell lines [117]; p21 expression detected in OS patient samples [118,119]; interacts with Runx2 to interrupt osteoblast differentiation in OS [120]	Normal development with no tumours detected at 7 months [121]; Spontaneous tumours detected at 16 months, predominantly soft-tissue sarcomas [122]; Soft tissue sarcoma detected in mice with deletions in WRN and p21 [88]
c-fos	No	Oncogene; transcription factor	Detection of c-fos in spontaneous & radiation-induced OS samples in mice [123]; Overexpression in human OS tumours, especially in relapsed and metastasised tumours [124,125]	Transgenic mice gave rise to OS [126,127]
Twist	Saethre-Chotzen Syndrome	Transcription factor, downstream of Runx2; transient loss in Twist is required in osteoblast differentiation [128]; Found to inhibit p53-modulated apoptosis through the interaction of ARF [129]	Found to be expressed in soft tissue sarcomas [129]; Twist found to be deleted or amplified in OS tumours [130,131]	Mice lacking the expression of Twist and APC gave rise to OS tumours [132]
Wnt signaling-pathway	Tooth agenesis, Colorectal Cancer, Anonychia [133,134]	Regulator of cell proliferation and differentation during embryonic development	Members of the Wnt pathway were detected in OS cell lines with suggested links to metastasis [135,136]	Inhibition of Wnt signaling (thru the use of DKK) in MSCs resulted in sarcoma formation [137]
WWOX	Eosphgeal Squamous Cell Carcinoma [138]	Oxidoreductase, located within fragile site locus [139]; potential tumour suppressor gene [140]	Absent or reduced WWOX expression detected in human OS samples [141]	OS was detected in juvenile wwox null mice [142]

### c-Fos

The overexpression of c-Fos was first noted in human OS tumour samples, particularly in metastasized tumours [124,125]. Its expression was also detected in mouse sporadic and radiation-induced OS [123]. In addition, genetically engineered mice that overexpressed c-Fos developed OS, thus suggestive of its role in OS pathogenesis [126,127]. However, the overexpression of c-Fos in humans is linked to fibrous dysplasia, of which less than 2% of patients develop OS [143,144]. Also, a recent study detected no change in c-Fos gene expression between human osteoblasts and OS tumours, which is in conflict with findings from Gamberi and Wu [66]. Therefore, the role of c-Fos in OS requires further studies to close the gap between transgenic mouse biology and human clinical studies.

### c-MYC

Amplification of the *c-MYC* gene is more prominent in Paget’s disease-related OS as compared to primary OS, although genetic rearrangement does not appear to be the cause [145,146]. Clinically, c-MYC expression levels in OS tumour samples was linked to resistance to methotrexate, with high c-MYC expression correlating to worse outcomes in OS patients [147].

A small cohort of transgenic mice developed OS when *c-MYC* expression was turned on with a tetracycline regulated transgene in haematopoietic cells [148]. The OS arising in these studies was most likely a result of ectopic expression of the transgene in osteoblastic cells. When *c-MYC* expression was inactivated by doxycycline administration, tumours transplanted into syngeneic mice regressed as OS cells differentiated into mature osteocytes [149]. In a subsequent report from the same group, the tumour regression from *c-MYC* inactivation in OS cells was attributed to the induction of senescence [150]. The development of OS was also reported in retrovirally transdcued *c-MYC*-overexpressing mesenchymal progenitor cells derived from *Ink4a/Arf* mutant mice [115].

### Osteoblast lineage restricted expression of Simian Virus 40 (SV40) T antigen

Antigens of the SV40 virus interact with and inactivate tumour suppressor genes including both Rb and p53 [151,152]. Interestingly, the SV40 gene was detected in a portion of human OS tumours, of which the sequence revealed viral integration in half of these tumours [153]. Early studies of transgenic mice that expressed SV40 antigens presented with OS and other tumours [154,155]. A recent study of mice that expressed the SV40 T antigen in mature osteoblasts using the osteocalcin promoter presented with bone tumours and were morbid by 21 weeks of age. This timeframe for tumour development is strikingly similar to that observed with *Osx*-Cre *p53*^*fl/fl*^*pRb*^*fl/fl*^ animals. The tumours in *Ocn*-SV40Tag animals were histologically confirmed as OS and possessed various levels of calcification. Also, the OS tumours metastasized at high frequency and were found predominantly in the lung and spleen [156].

Further analysis of tumours derived in this model revealed a recurrent genomic deletion of the *Prkar1a* gene [156]. Correspondingly, deletion of 1 allele of *Prkar1a* dramatically accelerated OS formation in mice with *Ocn*-SV40 T antigen with tumours arising within 5 weeks of birth. The analysis of human tumours found a subset of human OS also habour a *Prkar1a* deletion, demonstrating the power of mouse models to uncover new information into the complex genetics of human OS.

### Cell cycle genes: p15INK4b, p16INK4a

Several negative regulators of the G_1_-S cell cycle phase transition have been implicated in human OS. These fall into the “Rb pathway” and provide further support to the near obligate nature of this pathway disruption in the genesis of OS. p15INK4b was demonstrated to be repressed by c-MYC expression [157]. Mice deficient for *p15*^*INK4b*^ (along with *p14*^*ARF*^ and *p16*^*INK4a*^) developed a wide spectrum of cancers, including soft tissue sarcomas [111]. Genetic alterations were found in human patient-derived OS cell lines in the *p15*^*INK4b*^ locus [112]. Deletions of the *p16* genomic locus were apparent in samples from OS patients [158]. Loss of p16^INK4A^ expression was found in pediatric OS samples, with its expression level correlating to survival rates [114].

## Translating human cancer into animal models: issues & challenges

### Human cell lines vs animal models?

Experimental studies of OS have involved the use of cell lines and animal disease models [159,160]. However, cytogenetic complexity in human OS has confounded the efforts [161]. In particular, some human OS cell lines such as U2OS and SAOS-2 have been in use and passaged for many decades [162,163]. The extended passage and tissue culture can result in the acquisition of adaptive mutations from cell-culture conditions, as seen in long-term culturing of embryonic stem cells and lung cancer cell lines [164-166]. As such, the drift in gene expression signatures may make it less representative of the original tumour tissue and also lead to heterogeneity of the cell line populations held by different investigators [167,168]. The recent establishment and description of new OS cell lines opens up new avenues of study and hopefully improves the fidelity of tissue culture studies when referenced back to the human disease.

Murine and canine primary OS-derived cells have an advantage in this aspect. As a result of the relatively large amounts of primary, non-treated tumour tissue being available it is possible to establish early-passage cell lines for studies. Also, as mice on pure genetic backgrounds can be used, this will eliminate a significant source of intra-sample variation. The gene signatures from these lines would be expected to more closely mimic their primary tumour counterpart [165]. Also, the issues of over-passaging and culture-adaptation would be avoided as a result [169,170]. Most importantly, paired primary and metastatic disease samples from untreated mice can be isolated for robust comparisons of paired disease. This research aspect would not be readily possible from available human samples and canine OS cell lines.

The recent study in the identification of the Prkar1a gene performed by Khokha and colleagues highlights the power of genetically engineered murine models to gain new insights into human OS genetics [156]. In particular, the use of high-resolution comparative genomic hybridization (cGH) in primary tumours among other complementary analytical techniques was utilized in this project. This allows biologically relevant genetic changes during OS pathogenesis to be isolated, defined and validated from aneuploidy- associated “noise”. Such approaches coupled with the developed murine models may allow significant advances in our understanding of the complexity of OS.

The comparison of primary and metastatic disease from as many of these models as possible would be a novel approach to develop a better understanding of metastatic disease. This will be very useful for understanding the genetics and cell biology of metastatic OS, and the epigenetic processes that drive these mechanisms. The experimental approach focused on by analysis of paired primary and metastatic tumours and cell lines derived from the same animal should provide a strong basis for identifying key drivers of the progression and maintenance of metastatic disease. Such an approach could be a starting point to develop better therapeutic strategies for treating metastatic disease, the primary cause of mortality in OS patients.

### Different mouse models for different OS conditions

Various technological advancements have been incorporated into generating transgenic cancer mouse models. This includes germline & conditional knockouts, alleles bearing point mutations and tissue/region-specific gene expression [171,172]. These technologies have allowed for multiple paradigms in exploring targeted gene expression and its role in OS pathogenesis. For instance, the Cre-Lox system is widely used to turn off the expression of targeted genes [173]. The turning off of desired genes using Cre-Lox is most often an irreversible step and is useful for modelling OS related to the partial and complete loss of gene function. For instance, the occurrence of OS in mice with homozygous p53 and Rb deletions mimics the clinical scenario of patients with autosomal-dominant hereditary disorders as well as lesions found in the sporadic OS population [24,25].

The mouse models employed by two separate groups produced varying OS incidence rates, which was correlated with pRb and p53 status [24,25]. This observation is concordant with various sporadic-OS patient reports where allelic alterations for both genes were reported retrospectively [42,174-176]. The murine models have suggested strongly that deficiency for p53 is a strong initiating event for the development of OS and that disruption of the Rb pathway is a strongly synergistic mutation. The recent work from the Lees group provides an elegant model for the interaction and relative contribution of the p53 and pRb pathway mutations to the biological aspects of OS [56]. An unresolved question which will require analysis of human OS is to determine if the genetic alterations in OS could be different between sporadic and those associated with hereditary disorders.

An outstanding question is do mutations in all members of the p53 and Rb pathways contribute equally to tumour formation? For example, null mutation of the cyclin-dependent kinase *p27*^*Kip1*^, which results in deregulation of the “Rb pathway” did not result in OS in these mice [122,177]. When coupled with a *p53* mutation would *p27*^*Kip1*^ or *p21*^*Cip1*^ deficiency recapitulate all or only partial aspects of the loss of *Rb*? This is intriguing in light of the spectrum of mutations that have been reported in human OS. It provides an opportunity to compare mutations in distinct components of these pathways directly in the murine models that have been developed.

The emerging use of RNA interference (RNAi) in transgenic cancer models presents an exciting avenue to explore OS genetics and therapeutics. This is because the expression of targeted genes can be manipulated reversibly in a temporally controlled fashion to elucidate its biological purpose [178-180]. Also, this model provides the attractive prospect of exploring therapeutic target inhibition and resistance. As siRNA/shRNA represents a loss of function allele that are efficient but rarely complete this technology could be harnessed for the rapid and large scale *in vivo* screening of putative therapeutic targets. As small molecule inhibitors, like siRNA/shRNA, provide efficient but rarely complete target inactivation the testing of candidate therapeutic targets is highly suited to this approach.

### The OS cell of origin

The OS cell of origin has been widely discussed in the research literature. Its identity was proposed to be mesenchymal stem cells due to its potential to give rise to osteoblasts [181-183]. It also aligns with the notion that OS is differentiation-defective, due to the lack of terminally differentiated osteoblastic cells [120,184,185]. Identified by expression of Runx2, these mesenchymal progenitors are purported to be the source of OS initiating cells [186-189]. It is important to note that the cancer cell of origin is not necessarily related to the origin of the cancer stem cell [190]. Likewise, the OS cell of origin need not be mesenchymal stem cells, despite the various postulations suggesting this. In particular, the deletion of p53 in mesenchymal progenitor cells only yielded 61% of OS, with the rest being poorly differentiated soft-tissue sarcoma [55]. Also, soft-tissue sarcomas seem most likely to arise from mesenchymal stem/progenitor cells [191,192]. As the multipotent mesenchymal/skeletal stem cells can give rise to bone, cartilage and adipose cell lineages, perhaps it plays a more realistic purpose as a pan-sarcoma cell of origin.

**Figure 1:**
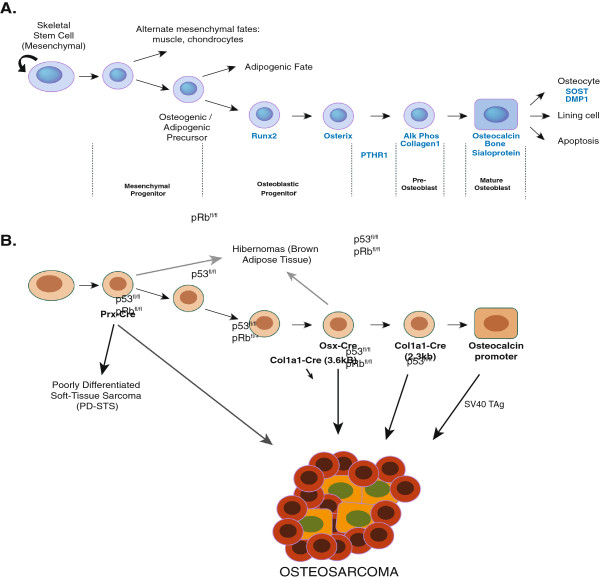
**Schematic representation of osteoblasticlineage commitment and differentiationfrom mesenchymal progenitors andthe models developed ingenetically engineered murine models.A**) Normal osteoblast development from mesenchymal stem cells. Genes associated with the commitment and differentiation of osteoblasts are listed along with an approximation of the developmental state of the osteoblastic cells. **B**) Using the differentiation schematic, the different Cre lines that have been described are in bold with an approximation of the putative cells expressing the Cre. The alleles that are disrupted (loss of function for p53 and pRb; over-expression for SV40TAg) are shown on the arrows. Other tumour types associated with the different models are also highlighted. For further information on these alleles see Table 2.

Data derived from a range of genetic approaches most strongly favours the OS cell of origin to be found within the committed osteoblast lineage. In particular, accumulating experimental evidence is most consistent with OS arising from the osteoblastic progenitor population [24,25]. For instance, the deletion of p53 in pre-osteoblasts and osteoblast progenitors resulted in significantly higher OS incidence rates than early multi-lineage potential cells (Figure 1 and Table 2). As osteoblast progenitors are more committed than their mesenchymal counterparts, this would correlate to decreased occurrence of other sarcoma types. As such, these studies strongly propose that OS arises from the osteoblast lineage-committed progenitor population and that the resulting tumour phenotype is a result of the accumulated genetic mutations that are present.

**Table 2:** **OS Incidence rates, murinegenotypes & its associatedcell lineages**

**Cell lineage**	**Genotype**	**OS penetrance**
Mesenchymal / Skeletal progenitors	*Prx1*-Cre-*p53*^*fl/fl*^	61% [55]; 62% [56]
	*Prx1*-Cre-*p53*^*fl/fl*^*-Rb*^*fl/+*^	92% [56]
	*Prx1*-Cre-*p53*^*fl/fl*^*-Rb*^*fl/fl*^	18% [55]; 29% [56]
Pre-Osteoblasts	*Osx*-Cre-*Rb*^*fl/fl*^	0% [24]; 0% [25]
	*Osx*-Cre-*p53*^*fl/fl*^	100% [24]; 100% [25]
	*Osx*-Cre-*p53*^*fl/fl*^*-Rb*^*fl/fl*^	53% [24]; 100% [25]
	*Osx*-Cre-*p53*^*fl/fl*^*-Rb*^*fl/+*^	72% [24]; 100% [25]
	*Col1*〈*13.6*-Cre-*p53*^*fl/fl*^	60% [193]
Osteoblasts	*Col1*〈*12.3*-Cre-*p53*^*fl/fl*^	85% [55]
	*Osteocalcin*-SV40 T antigen	100% [156]

### Metastatic disease – high fidelity and high penetrant models

The use of cancer mouse models with high penetrance allows a substantial population of mice with metastatic disease to be established. In particular, the mice generated by 3 separate groups developed OS with significant metastasis to soft tissues [24,25,156]. These models will be valuable in pre-clinical studies, as primary and metastasized tumours could be procured for the comparative studies. Advances in small animal imaging techniques such as μPET and μCT coupled with serology for alkaline phosphatase make possible the establishment of cohorts of animals with primary and a small metastatic disease burden. This strategy makes possible an assessment of therapeutic interventions in the context of primary and metastatic disease which are the most pressing clinical need. Longitudinal studies using such approaches would be an effective means to test and triage candidate therapeutic approaches in a controlled and reproducible manner. When coupled with xenografts of human material it may facilitate translation into rational clinical trials. Also, untreated paired tumour tissue will be useful as it is not readily collected in humans.

## Conclusion

Li-Fraumeni, Retinoblastoma and Rothmund-Thomson Syndrome are three human familial cancer syndromes that present with the strongest association to OS. Amongst sporadic OS, a wider range of genes and members of the p53 and Rb pathways are also implicated in OS pathogenesis. These mutations fulfill a range of the prerequisite requirements associated with the hallmarks of cancer, however the genes do not carry equal importance in tumour biology nor fully account for the pathogenesis of OS [194]. The integration of genetically engineered murine models based on familial human genetics of OS and additional experimental models such as the spontaneous OS arising in large breed dogs combine to form the basis of a preclinical platform that can serve to translate the extensive basic research efforts associated with OS to a clinically meaningful advantage. The use of primary human xenografts, in contrast to approaches using established human OS cell lines, adds an important component to the preclinical assessment phase of any new therapeutic options [195]. The underlying genetics in OS covers a wide spectrum, ranging from complete loss of gene function to hypomorphic mutations and gain of function. Various genetically modified mouse models of OS are now available and have demonstrated clearly that these are able to recapitulate the clinical spectrum of human OS.

## Abbreviations

BLM: Bloom; LFS: Li-Fraumeni Syndrome; L-MTP-PE: Liposomal Muramyl-Tripeptide Phosphatidyl Ethanolamine; Ocn: Osteocalcin; OS: Osteosarcoma; PD-STS: Poorly differentiated soft tissue sarcoma; Rb: Retinoblastoma; shRNA: Short hairpin RNA; siRNA: Small interfering RNA; SQSTM1: Sequestosome1; SV40: Simian Virus 40; RNAi: RNA interference; Tag: T antigen; Terc: Telomerase RNA Component; WRN: Werner.

## Competing interests

The authors would like to declare that there are no competing financial, professional or personal interests that might have influenced the performance or presentation of the work described in this manuscript.

## Authors' contributions

AJN, AJM, and EKB acquired data and drafted the manuscript. CRW conceived, participated in the design, coordination and helped to draft the manuscript. All authors read and approved the final manuscript.
